# A Cryo-EM Method to Solve Small Nucleic Acid Structures

**DOI:** 10.1063/4.0000953

**Published:** 2025-10-27

**Authors:** Holly L. Shultz, Evan R. Cramer, Aaron R. Robart

**Affiliations:** West Virginia University, Morgantown, West Virginia, USA

## Abstract

Nucleic acids adopt diverse structures that are essential to their biological function. Probing the structure-function relationship of nucleic acids provides critical insight into the principles governing molecular recognition and catalysis. DNAzymes are catalytically active DNA molecules created through in vitro selection. RNA-cleaving DNAzymes are promising candidates as therapeutics targeting disease-related mRNA. However, a major barrier to DNAzyme applications is the limited understanding of how DNAzyme structure governs catalytic activity. Structural methods often face technical challenges when applied to nucleic acids. In particular, the small size of DNAzymes (<20 kDa) poses significant limitations for cryo-EM analysis. Here, we present a broadly applicable cryo-EM strategy for visualizing DNAzyme structures using a protein scaffold. Applying this method to the well-characterized RNA-cleaving 10-23 DNAzyme, we obtained a detailed structural view of its catalytically relevant monomeric conformation. These results provide mechanistic insight and establish a pipeline for structural analysis of diverse DNAzymes. Ultimately, this will allow advancement of our general knowledge of how DNA structure promotes enzymatic function and facilitate the development of DNAzyme applications.

